# Supplementary material for: Immunopeptidomic Analysis of BoLA-I and BoLA-DR Presented Peptides from Theileria parva Infected Cells
Source: Vaccines (Basel). 2022 Nov 11;10(11):1907. doi: 10.3390/vaccines10111907 (PMC9699068; doi:10.3390/vaccines10111907)
Supplement: Supplementary file 1 [file vaccines-10-01907-s001.zip › Supplementary Data Captions.docx]

**Supplementary Data 1:** Summary statistics of the *T. parva* peptides eluted from samples subjected to BoLA-I (Supplementary Data 1.1) and BoLA-DR (Supplementary Data 1.2) elution in this study. For each sample summary data are provided for both the uncorrected *T. parva* peptidome (i.e. prior to removal of co-precipitants) and for the corrected *T. parva* peptidome (i.e. after removal of co-precipitants). The data includes the number of *T. parva* peptides, the percentage of the total peptidome (i.e. the combined number of bovine and *T. parva* derived peptides) that the number of *T. parva* peptides represents, the average (median) predicted % rank score (%RPS) of the *T. parva* peptides, the number and percentage of the *T. parva* peptides that were considered to be predicted binders (i.e. had a %RPS <2% for BoLA-I and <5% for BoLA-DR) and the average length of the *T. parva* peptides.

**Supplementary Data 2:** *T. parva* peptides derived by BoLA-I elution from (Supplementary Data 2.1) 2824TP, 5350TP, 2408TP, 2123TP, 5350TP_rpt, 2123TP_rpt, 641TP_rpt, 2824TP_rpt, 5072TP, 5072TP_rpt and BoLA-DR elution from (Supplementary Data 2.2) 2123TP_DR, 2824TP_DR, 495TP_DR, 5350TP_DR, 641TP_DR and 5072TP_DR. In Supplementary Data 2.1 for each peptide the amino acid sequence (column A), -10lgP score (B), peptide length (C), sample (D), number of spectra (E), MHC allele to which the peptide has the highest % rank binding score (F), core peptide sequence (G), % rank prediction score (H), accession number of the source protein (I), description of the source protein (J) and the peptide location within the source protein (K and L) are shown. (Equivalent data for 641TP, 1011TP and 2229TP samples are provided in Table I). In Supplementary Data 2.2 the same data is provided but in column I is shown the context of the peptides, with subsequent data shifted by 1 column.

**Supplementary Data 3:** Analysis of putative co-precipitants in the *T. parva* peptide datasets obtained from BoLA-I (BoLA-I dataset) and BoLA-DR (BoLA-DR dataset) elutions and a summary of the proteins that are sources of co-precipitants (Co-precipitating proteins). Co-precipitants were defined as overlapping peptides identified in multiple samples expressing non-matching MHC genotypes where one of more of the peptides were predicted to be non-MHC binders (defined as a % rank predicted score of >20%). Peptides designated as putative co-precipitants are identified in column M as YES; peptides removed as they were derived from either ribosomal proteins, histones or TpM_02g00758 are identified in column N as YES. Worksheets ‘MHCI dataset’ and ‘DR dataset’ provide the information from the combined BoLA-I and DR datasets respectively. The ‘Co-precipitating Proteins’ spreadsheet provides details of the proteins identified as the source of co-precipitating peptides. The number of peptides from each protein that were removed as putative co-precipitatants is shown.
